# Is PBPK useful to inform product label on managing clinically significant drug interactions mediated by cytokine release syndrome?

**DOI:** 10.1002/psp4.13185

**Published:** 2024-06-12

**Authors:** Xinyuan Zhang, Ping Zhao

**Affiliations:** ^1^ Quantitative Clinical Pharmacology Daiichi Sankyo, Inc. Basking Ridge New Jersey USA; ^2^ Bill & Melinda Gates Foundation Seattle Washington USA

Evaluating drug interactions caused by cytokine release syndrome (CRS) with PBPK (Physiologically Based Pharmacokinetic) modeling has been reported in some bispecific antibody regulatory submissions for 10 years. However, the published regulatory reviews and sponsors' analyses seem to disagree on the roles of PBPK modeling in regulatory decision‐making. In this editorial, we reviewed and provided our opinions on the FDA's current practice and sponsors' position in evaluating CRS‐mediated drug interactions. We discussed what has been done and what is lacking in the current PBPK approach assessing the CRS‐mediated drug interactions and proposed areas to bridge the gaps. And finally, we call to actions to improve the current practice toward a patient‐centric clinical pharmacology approach with more quantitative assessment and management of CRS‐mediated drug interactions.

The manuscript by Willemin et al.^1^ described the use of a PBPK approach to evaluate the effect of elevated IL‐6 following the treatment of teclistamab on the PK of CYP enzyme (1A2, 2C9, 2C19, 3A4, 3A5) substrates. This marks the 4th PBPK publication by CPT‐PSP of the effect of CRS as a result of biologics‐treatment on co‐medications that are CYP substrates, after blinatumomab,^2^ mosunetuzumab,^3^ and glofitamab.^4^ The scientific community and drug developers are using the PBPK modeling tool to study the effect of CRS on the PK and safety of co‐administered CYP substrate drugs. However, there seems to be a gap between the peer‐reviewed papers^1^, ^2^, ^3^, ^4^ and the regulatory evaluations^5^, ^6^, ^7^, ^8^ in terms of concluding the impact of PBPK predictions. In this editorial, we examine the gap and share our opinions on the value, expectation, and future of PBPK modeling in this specific area with the aim of increasing awareness, calling for enhanced predictive performance, and ultimately, achieving patient‐centric clinical pharmacology.

Cytokine release syndrome is characterized by the rapid release of pro‐inflammatory cytokines and immune cell activation. T cell‐engaging bispecific antibodies can cause transient release of cytokines that may potentially suppress CYP450 enzymes. Utilizing the PBPK modeling approach to evaluate the CRS‐mediated drug interactions in a regulatory submission can be traced back to the first FDA‐approved T‐cell‐engaging bispecific antibody, blinatumomab, in 2014.^5^ Over the past 10 years, a few additional T‐cell‐engaging bispecific antibodies were approved by FDA (mosunetuzumab, tebentafusp, teclistamab, epcoritamab, glofitamab, and talquetamab). We examined the FDA's biologics license application assessment packages, USPIs (United States Prescribing Information), and relevant PBPK publications to see how drug interactions mediated by CRS were evaluated and reported to healthcare professionals.

Among the seven programs (blinatumomab, mosunetuzumab, tebentafusp, teclistamab, epcoritamab, glofitamab, and talquetamab), no dedicated drug–drug interaction (DDI) study for CRS was conducted. Except for tebentafusp, six programs include “cautious periods” when the CRS‐mediated drug interactions may occur and ask for monitoring for toxicity of concomitant CYP substrates where minimal changes in concentration may lead to serious adverse reactions in Section 7 (Drug Interactions) of USPI. PBPK analyses were submitted for five programs (blinatumomab, mosunetuzumab, teclistamab, glofitamab, and talquetamab) to evaluate the duration and magnitude of CRS‐mediated drug interactions (Table 1). None of the PBPK evaluations was deemed adequate by the FDA. Therefore, it appears that the recommendation in Section 7 of USPI is based on the observed CRS period. We also noted that the delayed onset and offset effect of IL‐6 on CYP enzymes, and the enzyme turnover rate were considered by the FDA when recommending the “cautious period” for talquetamab.^9^

**TABLE 1:** Summary of drug interaction evaluation in FDA‐approved T‐cell‐engaging bispecific antibodies with PBPK analysis.

Generic Name/Trade Name/Year Approved/Pharmacologic class	FDA Conclusions on PBPK Model Adequacy and Rationale	Description of DDI Potential in USPI	Sponsor's Publications	Cytokines Assessed in Trials	CYPs Assessed in PBPK Model
Blinatumomab/Blincyto/2014/ bispecific CD19‐directed CD3 T‐cell engager	Conclusion: Inadequate^5^ Rationale: The PBPK model prediction cannot adequately address drug interaction potential of blinatumomab, as an exposure–response relationship between plasma IL‐6 levels and change in CYP activities in humans has not been established	The highest drug–drug interaction risk is during the first 9 days of the first cycle and the first 2 days of the second cycle.	Modeling IL‐6 profile: An IL‐6 model with zero‐order infusion and first‐order elimination kinetics were assumed to describe transient IL‐6 profile after dosing of biologics. Worst‐case investigation: Both the mean IL‐6 profile, and the IL‐6 profile in patient with the highest IL‐6 (*C*_max_ ~20,000 pg/mL) after dosing of biologics were investigated for CYP suppression potential.^2^	TNF‐α, IL‐2, IL‐6, IL‐8, IL‐10, IL‐12, IL‐4, IFN‐γ	3A4, 1A2, and 2C9
Mosunetuzumab‐axgb/Lunsumio/2022 /bispecific CD20‐directed CD3 T‐cell engager	Conclusion: Inadequate^6^ Rationale: PBPK analyses are inadequate to evaluate drug interaction potential of mosunetuzumab and duration of its effect on CYP3A substrates because exposure–response relationships between cytokines and CYP3A activity in human have not been established. The time course of the effects of cytokine release caused by antibody treatment on CYP enzyme activity in non‐RA patients has not been studied. The ability of PBPK modeling to predict the time course of the effect of IL‐6 on CYP3A activity cannot be assessed	Increased exposure of CYP450 substrates is more likely to occur after the first dose of LUNSUMIO on Cycle 1 Day 1 and up to 14 days after the second 60 mg dose on Cycle 2 Day 1 and during and after CRS.	Modeling IL‐6 profile: i.v. infusion (a zero‐order input rate, mg/h) of different hypothetical doses (mg) was used to model IL‐6 formation following different doses of mosunetuzumab. Worst‐case investigation: Assumed significant elevation of IL‐6 in gut as a result of mosunetuzumab treatment to account for suppression of gut CYP3A in addition to liver. Used a virtual cancer population for DDI simulations. Used both the mean IL‐6 profile and the profile close to the 95th percentile of the observed data (extreme IL‐6 levels up to 12,000 pg/mL).^3^	IL‐2, IL‐6, IL‐10, TNF‐α, IFN‐γ	3A
Teclistamab‐cqyv/Tecvayli /2022/bispecific B‐cell maturation antigen (BCMA)‐ directed CD3 T‐cell engager	Conclusion: Inadequate^8^ Rationale: FDA requested additional information during the review of the sponsor's evaluation of DDI potential, including a quantitative assessment of the effects of CRS on CYP enzyme activities. The PBPK model limitations include the lack of clinical drug interaction data for model verification, evaluation was limited to IL‐6 effects, and lack of sensitivity analysis on interaction parameters	The highest risk of drug–drug interaction is expected to occur from initiation of TECVAYLI step‐up dosing schedule up to 7 days after the first treatment dose and during and after CRS.	Modeling IL‐6 profile: i.v. infusion model with different IL‐6 doses to describe observed data in patients. Worst‐case investigation: Used IL‐6 profiles representing mean IL‐6 *C*_max_ and the highest IL‐6 *C*_max_ of 288 pg/mL observed in patients to simulate CYP suppression.^1^, ^10^	IL‐6, IL‐10, TNF‐α, IFN‐γ, and IL‐2R	1A2, 2C9, 2C19, 3A4/5
Glofitamab‐gxbm/Columvi/2023/bispecific CD20‐directed CD3 T‐cell engager	Conclusion: Inadequate^7^ Rationale: The exposure – response relationship between plasma IL‐6 levels and CYP activity has not been established in humans	Increased exposure of CYP substrates is more likely to occur after the first dose of COLUMVI on Cycle 1 Day 8 and up to 14 days after the first 30 mg dose on Cycle 2 Day 1 and during and after CRS.	Modeling IL‐6 profile: i.v. infusion model with different IL‐6 doses was used to generate low, medium, and high IL‐6 transient profiles after dosing of biologics and compared with observed data. Worst‐case investigation: Used the highest IL‐6 profile to simulate CYP suppression (*C*_max_ of IL‐6 ~2000 pg/mL).^4^	IL‐2, IL‐6, IL‐10, TNF‐α, IFN‐γ	1A2, 2C9, 3A4
Talquetamab‐tgvs/Talvey /2023/bispecific G protein‐coupled receptor family C group 5 member D (GPRCD5)‐directed CD3 T‐cell engager	Conclusion: Inadequate^9^ Rationale: The exposure – response relationship between plasma IL‐6 levels and changes in CYP activity in vivo has not been established in humans	Increased exposure of CYP substrates is more likely to occur from initiation of the TALVEY step‐up dosing schedule up to 14 days after the first treatment dose and during and after CRS.	Modeling IL‐6 profile: i.v. infusion model with different IL‐6 doses to describe observed data in patients. Worst‐case simulation: Predicted CYP suppression liability using IL‐6 profiles representing observed systemic median and the highest *C*_max_ (3682 pg/mL) in patients.^11^	IL‐6, IL‐10, TNF‐α, IFN‐γ, IL‐2R	1A2, 2C9, 2C19, 3A4/5

Abbreviations: *C*_max_, maximal concentration; i.v., intravenous; IFN, interferon; IL, interleukin; IL‐1RA, interleukin‐1 receptor antagonist; IL‐2R, interleukin‐2 receptor; TNF, tumor necrosis factor.

On the contrary, the sponsors appear to have high confidence in their PBPK model predictions of CRS‐mediated drug interactions.^1^, ^2^, ^3^, ^4^ The quantitative prediction results are also published on the product website to inform the healthcare providers regarding the exposure changes of concomitantly administered CYP substrates.^10^, ^11^

In our opinion, the current practice of informing Section 7 of USPI is not optimal and not a patient‐centric clinical pharmacology approach. In the following sections, we evaluate what has been done and what is lacking in the current PBPK analyses, and propose approaches to improve the confidence in the PBPK modeling and simulation, and eventually to better inform the USPI, healthcare providers, and patients on the risk of CRS‐mediated drug interactions.

Because detailed FDA assessments were often redacted, we have to assume that the same analyses published by the sponsors were submitted in BLA (Table 1). We found that sponsors' analyses were generally rigorous and risk‐based. All sponsors developed a fit‐for‐purpose PK model of IL‐6 to capture transient elevation of the cytokine after dosing of the biologic product, combined cytokine profiles with its CYP suppression mechanism and turnover of the CYP enzyme to predict the magnitude and duration of DDI. For all cases, worst‐case scenarios were explored using IL‐6 profile that represented patient(s) with the highest observed elevation following treatment of the biologics. In some cases, the effects of CRS on CYP3A in the gut,^1^, ^3^ co‐medications that may suppress IL‐6,^1^ and underlying disease (using virtual cancer population)^3^ were evaluated.

The rationales behind FDA's inadequate conclusions include the lack of an established exposure–response relationship between IL‐6 and CYP suppression as well as the time course of the interaction, the focus on evaluating the effect of IL‐6 on CYP substrates but no other cytokines (such as IL‐2, IL‐6, IL‐10, TNF‐α, IFN‐γ, etc.), and the use of data in patients with chronic autoimmune and inflammatory diseases to validate PBPK model for IL‐6.^5^, ^6^, ^7^, ^8^ The IL‐6 levels in those diseases are generally much lower compared with the IL‐6 levels in CRS (a few hundred vs. several thousand pg/mL reported by the sponsors^2^, ^3^, ^4^, ^5^, ^6^, ^7^), while the reported in vitro EC_50_ of IL‐6 against CYP3A4 activity was ~200 pg/mL in the absence of dexamethasone.^12^ The limitation that the time course of the CRS‐mediated inhibition effect on CYP enzymes has not been studied (clinically) in the non‐rheumatoid arthritis patients and the PBPK modeling may not capture the time course of recovery from the suppression effect, is a valid point. However, the transient nature of CRS and the number of CYP enzymes likely affected by elevated cytokines indeed make it difficult to design and conduct dedicated clinical studies to address CRS‐mediated drug interactions.

We believe that PBPK offers a useful alternative to the dedicated drug interaction studies, and the ultimate goal of PBPK modeling is to better inform the clinical use, such as removing the cautious languages if no true concerns or providing guidance on dosage adjustment when there is a real concern on drug interaction. To achieve this goal, the clinical pharmacology community should realize that the current practice of informing CRS‐mediated drug interaction risks is neither optimal nor patient‐centric. We wish to see one break the pattern of applying a PBPK modeling that the agency continuously considers inadequate, yet the drug interaction risk being‐based primarily on the CRS period without the context of CYP suppression potency in Section 7 of USPI. We also acknowledge that some additional work is needed and propose addressing the following scientific gaps.

- Higher cytokine levels: The transient cytokine levels in CRS are generally higher than those observed in patients with chronic immune and infectious diseases, and could be higher than in vitro EC_50_ values.^12^ The PBPK model was generally validated against drug interaction data obtained at lower cytokine concentrations. Evidence on the effect of cytokines at higher concentrations on CYP enzymes might be needed given that the in vivo EC_50_ values have not been estimated.
- The effect of other cytokines on the CYP enzyme activities: The in vivo data supporting a direct effect of IL‐2, IL‐8, IL‐10, IL‐17, TNF‐α, or IFN‐γ are inconclusive.^12^ The decision on which cytokine(s) to focus on and which CYPs to evaluate therefore should continue to be based on pharmacology principles. Analysis of CYP suppression by elevated IL‐6 can help guide the decision on other cytokines/CYPs.
- The patient populations beyond the differences in cytokine levels: Different patient populations may have disease‐specific baseline cytokine levels,which can change upon therapeutic interventions. This should be considered in a PBPK analysis.

Additional in vivo data might be needed to address the knowledge gaps. A recent PBPK publication evaluated the impact of elevated IL‐6 on CYP3A substrates in patients with COVID‐19 predicted DDI liability under the highest observed IL‐6 concentration of 4462 pg/mL, which might provide an additional dataset for model validation.^13^ Ideally, a dedicated drug interaction study could be conducted with appropriate design and data collection for relevant model validation. This can be considered as an one‐time investment to evaluate future (and confirm the past) CRS‐mediated drug interactions. Recognizing the challenge of conducting dedicated clinical studies to fully address transient nature of CRS and the number of CYPs affected, one can establish real‐world evidence detecting potential adverse effects related to elevated exposure of co‐medications as a result of much higher cytokine levels during CRS . Considering the number of T‐cell‐engaging bispecific antibodies in development, and the common pathway of the postulated drug interaction through CRS, it might be worthwhile for both the sponsors and regulators to collaborate on addressing these knowledge gaps and enhancing the predictability of PBPK.

To summarize, we commented on the current practice of how CRS‐mediated drug interaction risk is being communicated by both the Agency and the sponsors. We expressed our opinion that the current practice is not optimal, not patient‐centric, and lacks quantitative evaluation. We reviewed what has been done and what is lacking in the current PBPK modeling to evaluate the CRS‐mediated drug interactions. We identified a few gaps and discussed the approaches to achieve the goal of using PBPK to better inform the clinical use of concomitant medications in CRS events. We hope that soon, there will be a breakthrough in utilizing the quantitative approach to inform patients and healthcare providers about CRS‐mediated drug interaction risks.

## FUNDING INFORMATION

No funding was received for this work.

## CONFLICT OF INTEREST STATEMENT

The authors declared no competing interests for this work.

## DISCLAIMER

The views expressed in this editorial are those of the authors and do not represent the opinions of their employers.
